# Computational Fluid Dynamics as an Adjunctive Functional Assessment of Airway Narrowing After Pulmonary Artery Banding: A Case Report

**DOI:** 10.1002/rcr2.70749

**Published:** 2026-09-01

**Authors:** Saki Saijo, Mayu Noda, Yuta Arai, Konomi Moriwaki, Kota Miyoshi, Masaki Terazawa, Shotaro Otani, Kazuki Momota, Hiroki Sato, Takuya Takashima, Toshiyuki Nunomura, Manabu Ishihara, Taiga Itagaki, Tomonori Iwasaki, Jun Oto

**Affiliations:** ^1^ Department of Emergency and Critical Care Medicine, Institute of Biomedical Sciences Tokushima University Graduate School Tokushima Japan; ^2^ Department of Pediatric Dentistry, Institute of Biomedical Sciences Tokushima University Graduate School Tokushima Japan; ^3^ Department of Radiology, Institute of Biomedical Sciences Tokushima University Graduate School Tokushima Japan; ^4^ Department of Emergency and Critical Care Medicine Tokushima University Hospital Tokushima Japan; ^5^ Department of Emergency and Disaster Medicine Tokushima University Hospital Tokushima Japan; ^6^ Department of Infection Control and Prevention Tokushima University Hospital Tokushima Japan

**Keywords:** airway compression, computational fluid dynamics, congenital heart disease, infant, pulmonary artery banding

## Abstract

Airway compression is a recognized complication after pulmonary artery banding (PAB) in infants with congenital heart disease; however, conventional imaging primarily provides anatomical information. We report an infant with respiratory instability after PAB who underwent computational fluid dynamics (CFD) as an adjunctive functional assessment. After extubation, recurrent intercostal retractions and oxygen desaturation occurred during crying. Contrast‐enhanced CT and bronchoscopy revealed mild‐to‐moderate tracheobronchial narrowing caused by dilated pulmonary arteries without intrinsic obstruction. A patient‐specific CFD model simulated inspiratory airflow at 100 mL/s, approximating crying. Total airway resistance was markedly elevated (15 Pa/mL/s vs. approximately 0.07 Pa/mL/s in healthy infants), with pronounced airflow asymmetry and reduced flow to the right main bronchus, suggesting functional impairment despite relatively preserved airway lumens. These findings were considered complementary to the overall clinical assessment, rather than an independent surgical indication. The patient's symptoms resolved after pulmonary artery plication and ventricular septal defect closure.

## Graphical abstract

An infant developed respiratory instability after pulmonary artery banding despite only mild‐to‐moderate tracheobronchial narrowing on computed tomography and bronchoscopy. Patient‐specific computational fluid dynamics revealed markedly elevated airway resistance (15 Pa/mL/s) and severe airflow asymmetry, with only 0.04% of the simulated inspiratory flow entering the right main bronchus.

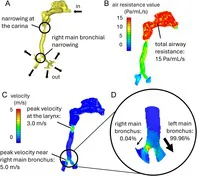

## 1 Introduction

Airway narrowing due to extrinsic compression is a recognized complication of pulmonary artery banding (PAB), particularly when postoperative pulmonary artery dilation impinges on the tracheobronchial tree [1, 2, 3]. Computed tomography (CT) and bronchoscopy provide anatomical information [3, 4], whereas computational fluid dynamics (CFD) can quantify airflow distribution and airway resistance using CT‐based geometry [5]. We report an infant with airway compression after PAB, in whom CFD provided an adjunctive functional assessment.

## 2 Case Report

A male infant with ventricular septal defect (VSD), pulmonary hypertension and dilated cardiomyopathy (DCM) underwent PAB at 3 months and 20 days of age. Height and weight were 55.1 cm and 4.14 kg, respectively. Preoperative echocardiography showed a reduced systolic function: left ventricular ejection fraction, 43%; global longitudinal strain, −12.3%. The left ventricular end‐diastolic diameter was 27.7 mm, corresponding to a z‐score of +0.9, which indicated mild left ventricular enlargement. Comprehensive evaluations supported the clinical diagnosis of mild DCM. A perimembranous VSD was 11.6 mm in diameter, with an effective diameter of 4.2 mm due to partial restriction by a membranous septal aneurysm. The Qp/Qs was 2.45–2.80, pulmonary arterial pressure was 79/39 mmHg and pulmonary vascular resistance was 4.89 Wood units m^2^. Because primary VSD closure was considered high risk owing to low body weight, pulmonary hypertension, impaired left ventricular systolic function and limited cardiac reserve, PAB was selected as a staged procedure. Preoperative respiratory symptoms or abnormal breathing patterns were absent, and a retrospective preoperative CT review showed no tracheobronchial compression.

PAB was performed with a circumference of 28 mm, corresponding to a nominal diameter of 8.9 mm. Postoperative echocardiography showed a peak banding‐site velocity of 2.27 m/s, corresponding to a pressure gradient of 21 mmHg. He was extubated on postoperative day (POD) 23. Recurrent intercostal retractions and desaturation developed during crying, with transient decreases to the mid‐80% range despite high‐flow nasal cannula therapy.

**FIGURE 1:**
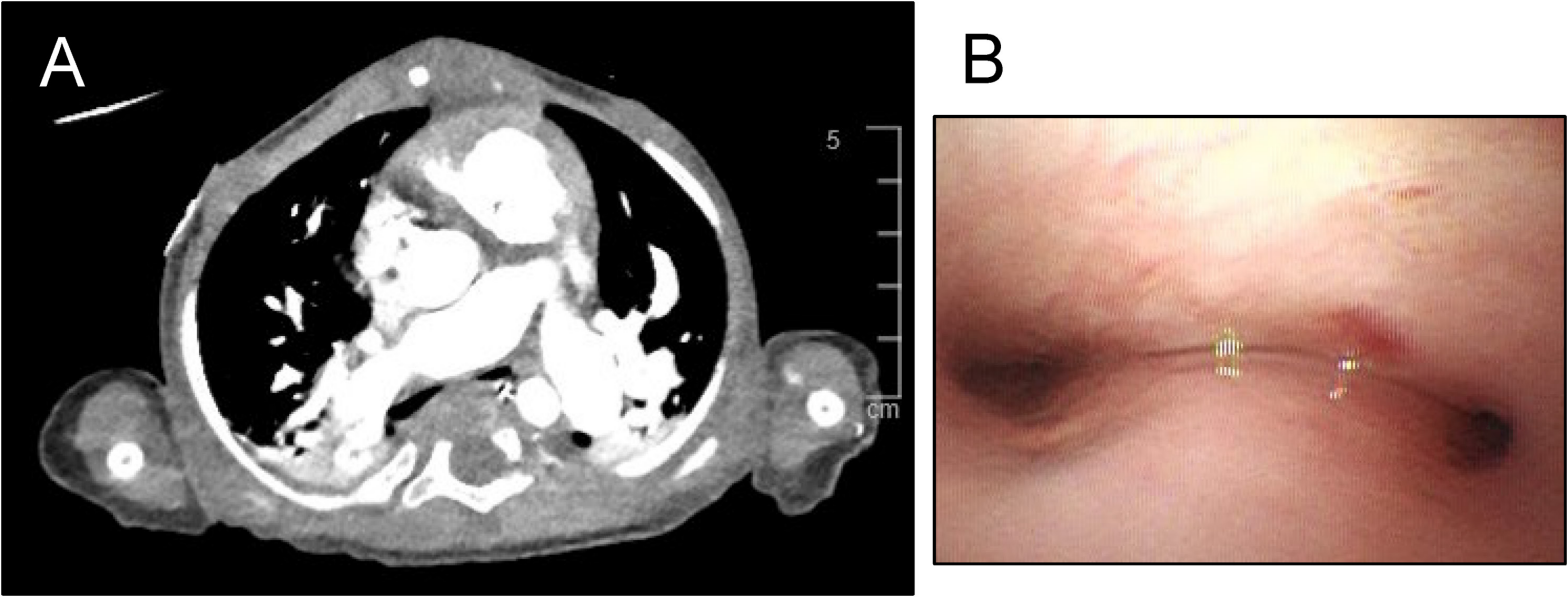
Imaging findings demonstrating tracheal bifurcation compression. (A) Contrast‐enhanced computed tomography showing narrowing of the tracheal bifurcation caused by extrinsic compression from dilated pulmonary arteries. (B) A bronchoscopic image revealing stenosis at the carina.

Contrast‐enhanced CT on POD 36 revealed mild‐to‐moderate narrowing of the tracheal bifurcation caused by dilated pulmonary arteries distal to the PAB site, without intrinsic obstruction or vascular anomalies (Figure 1A). Flexible bronchoscopy on POD 40 under intubation and controlled ventilation confirmed mild narrowing of the carina and main bronchi (Figure 1B), but dynamic airway behaviour could not be assessed.

**FIGURE 2:**
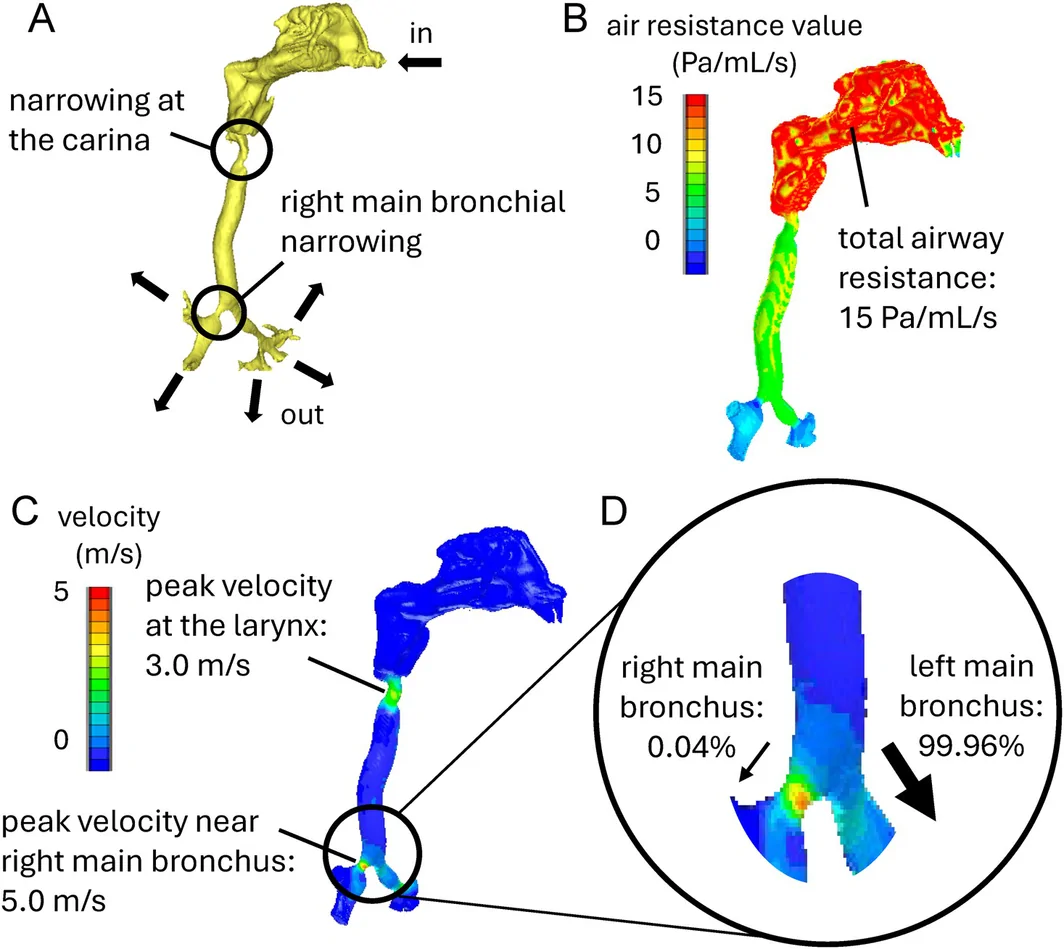
Patient‐specific computational fluid dynamics analysis demonstrating functional severity of airway narrowing. (A) Three‐dimensional reconstructed airway model derived from contrast‐enhanced computed tomography. The compressed tracheal bifurcation and right main bronchus are indicated. Simulations were performed under inspiratory conditions, with airflow entering from the nasal cavity and exiting through both main bronchi. (B) Distribution of airway resistance values at each airway segment during inspiration. The total airway resistance was markedly elevated at 15 Pa/mL/s, indicating substantial functional airflow limitation despite only mild to moderate anatomical narrowing on CT and bronchoscopy. (C) Airflow velocity distribution during inspiration, showing localized flow acceleration at the laryngeal level (maximum velocity, 3.0 m/s) and near the right main bronchus (maximum velocity, 5.0 m/s). (D) Simulated inspiratory airflow distribution showed marked asymmetry, with 99.96% of total flow entering the left main bronchus and only 0.04% entering the right main bronchus. These findings indicate severe functional impairment under high‐flow inspiratory conditions.

A patient‐specific three‐dimensional airway model was reconstructed using CT images (Figure 2A). A CFD analysis (Phoenics, CHAM Japan) simulated inspiratory airflow at 100 mL/s, approximating crying. Total airway resistance was markedly elevated (15 Pa/mL/s) relative to control infants (~0.07 Pa/mL/s: Figure 2B) [5]. The maximum inspiratory velocity reached 3.0 m/s at the larynx and 5.0 m/s near the right main bronchus (Figure 2C), with markedly reduced airflow to the right main bronchus (0.04% of total flow) (Figure 2D).

Although CT and bronchoscopy showed only mild‐to‐moderate luminal narrowing, recurrent desaturation and retraction which occurred when crying suggested clinically relevant airway compromise. CFD supported functional significance. Based on persistent respiratory instability, imaging, bronchoscopic findings and multidisciplinary assessment, surgery was planned. Pulmonary artery plication was performed to relieve the compression. Although initially considered high risk, primary VSD closure was performed to eliminate the left‐to‐right shunt and reduce pulmonary overcirculation and pulmonary artery dilation. We did not perform great vessel suspension or isolated PAB adjustment. The patient remained intubated and underwent pulmonary artery plication and VSD closure the following day.

A histopathological examination of the pulmonary artery wall revealed focal medial degeneration and small necrotic foci without inflammatory infiltration. Elastica van Gieson staining revealed a preserved vascular wall architecture with mild focal elastic fibre disruption. No myxomatous degeneration or developmental abnormalities were identified, thus suggesting secondary acute degenerative changes rather than primary structural abnormalities.

Postoperatively, veno‐arterial extracorporeal membrane oxygenation was required for refractory respiratory and cardiac dysfunction but was discontinued on POD 8. The patient was extubated on POD 33, and retractions and desaturation during crying resolved. The patient was discharged from the ICU on POD 41.

## 3 Discussion

Airway compression by cardiovascular structures is underrecognized in infants with congenital heart disease [2, 3]. After PAB, pulmonary artery dilation may compress the trachea or main bronchi [1]. Deterioration occurred during crying, suggesting that increased inspiratory flow unmasked the functional limitations. CT and bronchoscopy identified structural narrowing but did not quantify its physiological impacts. CFD showed markedly elevated airway resistance and airflow asymmetry, which helped explain the symptoms disproportionate to the anatomical narrowing.

This case should not be interpreted as showing that CFD can define an operative threshold or replace clinical evaluation. The course was atypical, and surgery was based on comprehensive judgement rather than CFD alone. Nevertheless, CFD provides functional information not captured by static anatomical assessment and may be useful when symptoms are disproportionate to imaging findings.

This study had some limitations. CFD uses simplified boundary conditions, rigid airway walls and static CT geometry and cannot reproduce dynamic airway behaviour during respiration or crying. Bronchoscopy was performed under intubation and controlled ventilation; dynamic changes during spontaneous breathing were not assessed. Unrecognized tracheomalacia or airway vulnerability cannot be excluded. The simulated flow rate approximated crying but was not patient‐specific, and the absolute resistance values should be interpreted cautiously. As this is a single atypical case, the findings are not generalizable and cannot be used to establish surgical thresholds. Postoperative CFD was unavailable because follow‐up CT was not performed. CFD requires CT‐based reconstruction and may not be feasible in unstable patients. Further cases are needed to clarify CFD's contribution to acute treatment decisions.

## Funding

This work was supported by JSPS KAKENHI Grant Number JP25K19876.

## Consent

The authors declare that written informed consent was obtained for the publication of this manuscript and accompanying images using the form provided by the Journal.

## Conflicts of Interest

The authors declare no conflicts of interest.

## Data Availability

The data that support the findings of this study are available from the corresponding author upon reasonable request.
